# 2-[(*E*)-(2-Chloro­phen­yl)imino­meth­yl]-6-methyl­phenol

**DOI:** 10.1107/S1600536810033969

**Published:** 2010-08-28

**Authors:** Peihua Zhu, Jiemei Yu, Hongyan Wang, Chunlai Zhang, Dongming Yang

**Affiliations:** a School of Chemistry and Chemical Engineering, University of Jinan, Jinan 250022, People’s Republic of China

## Abstract

The title compound, C_14_H_12_ClNO, a Schiff base derived from 3-methyl­salicyl­aldehyde, crystallizes in the phenol–imine tautomeric form with an *E* conformation for the imine functionality. The mol­ecule is not planar, the dihedral angle between the aromatic rings being 36.38 (5)°. The hy­droxy H atom is involved in a strong intra­molecular O—H⋯N hydrogen bond, generating an *S*(6) ring.

## Related literature

For background information and applications of Schiff base complexes, see: Barton & Ollis (1979 ▶); Layer (1963 ▶); Ingold (1969 ▶); Cohen *et al.* (1964 ▶); Henrici-Olive & Olive (1984 ▶); Garnovskii *et al.* (1993 ▶). For related structures, see: Köysal *et al.* (2007 ▶); Kılıç *et al.* (2009 ▶); Şahin *et al.* (2009 ▶).
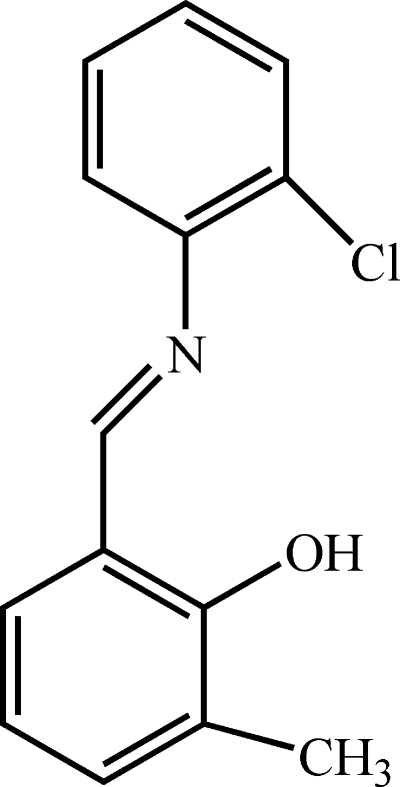


## Experimental

### 

#### Crystal data

C_14_H_12_ClNO
*M* *_r_* = 245.70
Orthorhombic, 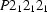

*a* = 7.8318 (14) Å
*b* = 11.693 (2) Å
*c* = 13.250 (2) Å
*V* = 1213.4 (4) Å^3^
*Z* = 4
Mo *K*α radiation
μ = 0.30 mm^−1^
*T* = 293 K
0.21 × 0.11 × 0.06 mm

#### Data collection

Bruker APEXII CCD area-detector diffractometer
Absorption correction: multi-scan (*SADABS*; Sheldrick, 2003 ▶) *T*_min_ = 0.940, *T*_max_ = 0.982
6803 measured reflections
2477 independent reflections
1486 reflections with *I* > 2σ(*I*)
*R*_int_ = 0.070

#### Refinement

*R*[*F* ^2^ > 2σ(*F* ^2^)] = 0.051
*wR*(*F* ^2^) = 0.096
*S* = 1.00
2477 reflections
158 parameters
1 restraint
H atoms treated by a mixture of independent and constrained refinement
Δρ_max_ = 0.15 e Å^−3^
Δρ_min_ = −0.14 e Å^−3^
Absolute structure: Flack (1983 ▶), 1034 Friedel pairs
Flack parameter: −0.06 (9)

### 

Data collection: *APEX2* (Bruker, 2004 ▶); cell refinement: *SAINT-Plus* (Bruker, 2001 ▶); data reduction: *SAINT-Plus*; program(s) used to solve structure: *SHELXS97* (Sheldrick, 2008 ▶); program(s) used to refine structure: *SHELXL97* (Sheldrick, 2008 ▶); molecular graphics: *SHELXTL* (Sheldrick, 2008 ▶); software used to prepare material for publication: *SHELXTL*.

## Supplementary Material

Crystal structure: contains datablocks global, I. DOI: 10.1107/S1600536810033969/zq2056sup1.cif

Structure factors: contains datablocks I. DOI: 10.1107/S1600536810033969/zq2056Isup2.hkl

Additional supplementary materials:  crystallographic information; 3D view; checkCIF report

## Figures and Tables

**Table 1:** Hydrogen-bond geometry (Å, °)

*D*—H⋯*A*	*D*—H	H⋯*A*	*D*⋯*A*	*D*—H⋯*A*
O1—H1⋯N1	0.87 (1)	1.84 (2)	2.615 (3)	148 (3)

**Fig. 1.**
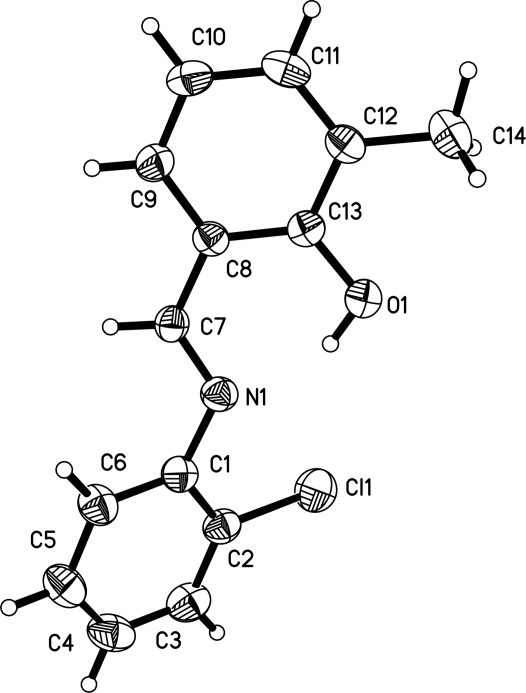
The molecular structure of the title compound, with the atom-numbering scheme and 25% probability displacement ellipsoids for the non-H atoms.

## supplementary crystallographic information

### Comment

Schiff bases are used as starting materials in the synthesis of important drugs (Layer, 1963; Ingold, 1969). A large number of Schiff bases and their complexes have been studied for their interesting and important properties, *e.g.* catalytic activity (Henrici-Olive & Olive, 1984), photochromic properties (Cohen *et al.*, 1964), biological activity (Barton *et al.*, 1979). On the other hand, Schiff base ligands play a vital role in coordination chemistry due to their metal binding ability (Garnovskii *et al.*, 1993).

The structure of the title compound is shown in Fig. 1. The C7═N1 double bond of 1.283 (3) Å is slightly longer than the literature values found in similar structures (Köysal *et al.*, 2007; Kılıç *et al.,* 2009; Şahin *et al.,* 2009) in the range of 1.262 (8)-1.279 (3) Å. The title molecule is not planar with a dihedral angle between the aromatic rings C1/C6 and C8/C13 of 36.38 (5) °. The imino group is coplanar with the hydroxyphenyl ring with the torsion angle C13—C8—C7—N1 of 1.6 (4) °.

The molecular structure is stabilized by a strong intramolecular O—H···N hydrogen bond.

### Experimental

A solution of 3-methylsalicylaldehyde (0.0681 g, 0.5 mmol) in ethanol (10 ml) was added to a solution of 2-chlorobenzenamine (0.0638 g, 0.5 mmol) in ethanol (20 ml). The reaction mixture was stirred for 2 h under reflux. Single crystals suitable for a X-ray analysis were obtained from ethanol by slow evaporation (0.0749 g, 61%).

### Refinement

The H atom bounded to O1 was located in the difference Fourier map and freely refined with *U*_iso_(H) = 1.2*U*_eq_ (O). All other H atoms were placed in calculated positions and refined using a riding-model approximation with C—H = 0.93 Å and *U*_iso_(H) = 1.2*U*_eq_(C) for aromatic H atoms, and with C—H = 0.96 Å and *U*_iso_(H) = 1.5*U*_eq_(C) for methyl H atoms.

### Crystal data

**Table:**

C_14_H_12_ClNO	*F*(000) = 512
*M**_r_* = 245.70	*D*_x_ = 1.345 Mg m^−^^3^
Orthorhombic, *P*2_1_2_1_2_1_	Mo *K*α radiation, λ = 0.71073 Å
Hall symbol: P 2ac 2ab	Cell parameters from 1163 reflections
*a* = 7.8318 (14) Å	θ = 3.1–28.8°
*b* = 11.693 (2) Å	µ = 0.30 mm^−^^1^
*c* = 13.250 (2) Å	*T* = 293 K
*V* = 1213.4 (4) Å^3^	Needle, yellow
*Z* = 4	0.21 × 0.11 × 0.06 mm

### Data collection

**Table:**

Bruker APEXII CCD area-detector diffractometer	2477 independent reflections
Radiation source: fine-focus sealed tube	1486 reflections with *I* > 2σ(*I*)
graphite	*R*_int_ = 0.070
φ and ω scans	θ_max_ = 26.4°, θ_min_ = 3.1°
Absorption correction: multi-scan (*SADABS*; Sheldrick, 2003)	*h* = −9→9
*T*_min_ = 0.940, *T*_max_ = 0.982	*k* = −14→14
6803 measured reflections	*l* = −16→16

### Refinement

**Table:**

Refinement on *F*^2^	Secondary atom site location: difference Fourier map
Least-squares matrix: full	Hydrogen site location: inferred from neighbouring sites
*R*[*F*^2^ > 2σ(*F*^2^)] = 0.051	H atoms treated by a mixture of independent and constrained refinement
*wR*(*F*^2^) = 0.096	*w* = 1/[σ^2^(*F*_o_^2^) + (0.0358*P*)^2^ + 0.0414*P*] where *P* = (*F*_o_^2^ + 2*F*_c_^2^)/3
*S* = 1.00	(Δ/σ)_max_ < 0.001
2477 reflections	Δρ_max_ = 0.15 e Å^−^^3^
158 parameters	Δρ_min_ = −0.14 e Å^−^^3^
1 restraint	Absolute structure: Flack (1983), 1034 Friedel pairs
Primary atom site location: structure-invariant direct methods	Flack parameter: −0.06 (9)

### Special details

**Table:**

Geometry. All e.s.d.'s (except the e.s.d. in the dihedral angle between two l.s. planes) are estimated using the full covariance matrix. The cell e.s.d.'s are taken into account individually in the estimation of e.s.d.'s in distances, angles and torsion angles; correlations between e.s.d.'s in cell parameters are only used when they are defined by crystal symmetry. An approximate (isotropic) treatment of cell e.s.d.'s is used for estimating e.s.d.'s involving l.s. planes.
Refinement. Refinement of *F*^2^ against ALL reflections. The weighted *R*-factor *wR* and goodness of fit *S* are based on *F*^2^, conventional *R*-factors *R* are based on *F*, with *F* set to zero for negative *F*^2^. The threshold expression of *F*^2^ > σ(*F*^2^) is used only for calculating *R*-factors(gt) *etc*. and is not relevant to the choice of reflections for refinement. *R*-factors based on *F*^2^ are statistically about twice as large as those based on *F*, and *R*- factors based on ALL data will be even larger.

### Fractional atomic coordinates and isotropic or equivalent isotropic displacement parameters (Å^2^)

**Table:**

	*x*	*y*	*z*	*U*_iso_*/*U*_eq_	
Cl1	−0.30427 (11)	−0.84282 (9)	−0.18201 (8)	0.0873 (3)	
C11	−0.2023 (4)	−0.5269 (3)	−0.6436 (3)	0.0609 (9)	
H11	−0.1358	−0.4949	−0.6945	0.073*	
C2	−0.6147 (5)	−0.8244 (3)	−0.0959 (3)	0.0673 (9)	
H2	−0.5727	−0.8740	−0.0469	0.081*	
C4	−0.8405 (4)	−0.7124 (3)	−0.1622 (3)	0.0704 (10)	
H4	−0.9518	−0.6851	−0.1577	0.085*	
C13	−0.2304 (4)	−0.5959 (2)	−0.4763 (2)	0.0460 (7)	
N1	−0.4592 (3)	−0.68266 (18)	−0.32559 (19)	0.0510 (6)	
C8	−0.4038 (3)	−0.6210 (2)	−0.4942 (2)	0.0446 (7)	
C5	−0.7384 (3)	−0.6793 (3)	−0.2418 (3)	0.0600 (8)	
H5	−0.7817	−0.6305	−0.2910	0.072*	
C1	−0.5125 (4)	−0.7905 (2)	−0.1741 (2)	0.0546 (8)	
C3	−0.7793 (5)	−0.7849 (3)	−0.0899 (3)	0.0726 (10)	
H3	−0.8492	−0.8075	−0.0368	0.087*	
C9	−0.4702 (4)	−0.5986 (2)	−0.5894 (2)	0.0568 (8)	
H9	−0.5835	−0.6167	−0.6030	0.068*	
C7	−0.5128 (3)	−0.6658 (2)	−0.4159 (2)	0.0476 (7)	
H7	−0.6257	−0.6830	−0.4313	0.057*	
C12	−0.1285 (3)	−0.5488 (2)	−0.5514 (2)	0.0509 (8)	
C6	−0.5706 (3)	−0.7186 (2)	−0.2491 (2)	0.0495 (7)	
C10	−0.3721 (4)	−0.5507 (3)	−0.6631 (2)	0.0638 (9)	
H10	−0.4190	−0.5342	−0.7260	0.077*	
C14	0.0568 (4)	−0.5250 (3)	−0.5305 (3)	0.0781 (11)	
H14C	0.1143	−0.5953	−0.5150	0.117*	
H14A	0.0662	−0.4737	−0.4742	0.117*	
H14B	0.1081	−0.4907	−0.5889	0.117*	
O1	−0.1569 (2)	−0.61668 (18)	−0.38591 (17)	0.0618 (6)	
H1	−0.233 (3)	−0.648 (3)	−0.3472 (19)	0.074*	

### Atomic displacement parameters (Å^2^)

**Table:**

	*U*^11^	*U*^22^	*U*^33^	*U*^12^	*U*^13^	*U*^23^
Cl1	0.0762 (5)	0.1058 (7)	0.0799 (7)	0.0203 (5)	−0.0073 (5)	0.0146 (6)
C11	0.072 (2)	0.0524 (19)	0.058 (2)	0.0033 (17)	0.0238 (19)	0.0008 (17)
C2	0.089 (3)	0.054 (2)	0.059 (2)	−0.0124 (19)	−0.002 (2)	0.0131 (19)
C4	0.064 (2)	0.065 (2)	0.083 (3)	−0.0066 (17)	0.016 (2)	0.000 (2)
C13	0.0515 (19)	0.0393 (16)	0.0471 (19)	0.0069 (13)	0.0008 (15)	−0.0042 (15)
N1	0.0543 (13)	0.0510 (14)	0.0477 (16)	−0.0051 (11)	0.0029 (14)	0.0016 (14)
C8	0.0473 (18)	0.0412 (17)	0.0452 (19)	0.0026 (12)	0.0044 (15)	−0.0075 (15)
C5	0.0581 (18)	0.0538 (19)	0.068 (2)	−0.0021 (15)	0.0058 (17)	0.0120 (18)
C1	0.0629 (18)	0.0511 (16)	0.0499 (19)	−0.0042 (15)	−0.0015 (18)	−0.0013 (18)
C3	0.087 (3)	0.064 (2)	0.067 (2)	−0.0186 (19)	0.022 (2)	0.002 (2)
C9	0.0564 (17)	0.0652 (18)	0.049 (2)	0.0009 (15)	−0.0027 (17)	−0.0051 (19)
C7	0.0468 (15)	0.0460 (16)	0.050 (2)	−0.0023 (14)	−0.0025 (16)	−0.0025 (17)
C12	0.0527 (17)	0.0432 (16)	0.057 (2)	0.0023 (14)	0.0098 (18)	−0.0034 (16)
C6	0.0539 (18)	0.0423 (16)	0.0523 (19)	−0.0094 (13)	0.0014 (16)	−0.0013 (17)
C10	0.079 (2)	0.071 (2)	0.042 (2)	0.0057 (18)	0.0028 (18)	−0.0004 (19)
C14	0.057 (2)	0.085 (2)	0.093 (3)	−0.0041 (17)	0.0114 (19)	0.004 (2)
O1	0.0516 (12)	0.0784 (16)	0.0554 (15)	−0.0011 (11)	−0.0025 (11)	0.0044 (12)

### Geometric parameters (Å, °)

**Table:**

Cl1—C1	1.745 (3)	C8—C9	1.388 (4)
C11—C12	1.376 (4)	C8—C7	1.442 (4)
C11—C10	1.384 (4)	C5—C6	1.396 (4)
C11—H11	0.9300	C5—H5	0.9300
C2—C1	1.369 (4)	C1—C6	1.378 (4)
C2—C3	1.372 (4)	C3—H3	0.9300
C2—H2	0.9300	C9—C10	1.363 (4)
C4—C3	1.366 (4)	C9—H9	0.9300
C4—C5	1.379 (4)	C7—H7	0.9300
C4—H4	0.9300	C12—C14	1.503 (4)
C13—O1	1.351 (3)	C10—H10	0.9300
C13—C12	1.389 (4)	C14—H14C	0.9600
C13—C8	1.410 (4)	C14—H14A	0.9600
N1—C7	1.283 (3)	C14—H14B	0.9600
N1—C6	1.402 (3)	O1—H1	0.87 (3)
			
C12—C11—C10	122.2 (3)	C2—C3—H3	120.1
C12—C11—H11	118.9	C10—C9—C8	121.2 (3)
C10—C11—H11	118.9	C10—C9—H9	119.4
C1—C2—C3	119.7 (3)	C8—C9—H9	119.4
C1—C2—H2	120.1	N1—C7—C8	122.2 (3)
C3—C2—H2	120.1	N1—C7—H7	118.9
C3—C4—C5	120.5 (3)	C8—C7—H7	118.9
C3—C4—H4	119.8	C11—C12—C13	117.9 (3)
C5—C4—H4	119.8	C11—C12—C14	122.3 (3)
O1—C13—C12	117.5 (3)	C13—C12—C14	119.8 (3)
O1—C13—C8	121.4 (3)	C1—C6—C5	117.5 (3)
C12—C13—C8	121.1 (3)	C1—C6—N1	119.9 (3)
C7—N1—C6	121.1 (2)	C5—C6—N1	122.5 (3)
C9—C8—C13	118.3 (3)	C9—C10—C11	119.3 (3)
C9—C8—C7	120.0 (3)	C9—C10—H10	120.3
C13—C8—C7	121.7 (3)	C11—C10—H10	120.3
C4—C5—C6	120.4 (3)	C12—C14—H14C	109.5
C4—C5—H5	119.8	C12—C14—H14A	109.5
C6—C5—H5	119.8	H14C—C14—H14A	109.5
C2—C1—C6	122.0 (3)	C12—C14—H14B	109.5
C2—C1—Cl1	119.4 (3)	H14C—C14—H14B	109.5
C6—C1—Cl1	118.6 (2)	H14A—C14—H14B	109.5
C4—C3—C2	119.9 (3)	C13—O1—H1	108 (2)
C4—C3—H3	120.1		
			
O1—C13—C8—C9	−179.2 (2)	C10—C11—C12—C14	178.9 (3)
C12—C13—C8—C9	0.7 (4)	O1—C13—C12—C11	−179.7 (2)
O1—C13—C8—C7	2.5 (4)	C8—C13—C12—C11	0.4 (4)
C12—C13—C8—C7	−177.6 (2)	O1—C13—C12—C14	1.0 (4)
C3—C4—C5—C6	−0.6 (4)	C8—C13—C12—C14	−178.9 (3)
C3—C2—C1—C6	−1.1 (5)	C2—C1—C6—C5	1.1 (4)
C3—C2—C1—Cl1	−179.1 (3)	Cl1—C1—C6—C5	179.1 (2)
C5—C4—C3—C2	0.6 (5)	C2—C1—C6—N1	177.5 (3)
C1—C2—C3—C4	0.2 (5)	Cl1—C1—C6—N1	−4.5 (3)
C13—C8—C9—C10	−1.8 (4)	C4—C5—C6—C1	−0.2 (4)
C7—C8—C9—C10	176.5 (3)	C4—C5—C6—N1	−176.6 (3)
C6—N1—C7—C8	175.4 (2)	C7—N1—C6—C1	146.1 (3)
C9—C8—C7—N1	−176.7 (3)	C7—N1—C6—C5	−37.6 (4)
C13—C8—C7—N1	1.6 (4)	C8—C9—C10—C11	1.8 (5)
C10—C11—C12—C13	−0.4 (4)	C12—C11—C10—C9	−0.7 (5)

### Hydrogen-bond geometry (Å, °)

**Table:**

*D*—H···*A*	*D*—H	H···*A*	*D*···*A*	*D*—H···*A*
O1—H1···N1	0.87 (1)	1.84 (2)	2.615 (3)	148 (3)

## References

[bb1] Barton, D. & Ollis, W. D. (1979). *Comprehensive Organic Chemistry*, Vol 2. Oxford: Pergamon Press.

[bb2] Bruker (2001). *SAINT-Plus* Bruker AXS Inc., Madison, Wisconsin, USA.

[bb3] Bruker (2004). *APEX2* Bruker AXS Inc., Madison, Wisconsin, USA.

[bb4] Cohen, M. D., Schmidt, G. M. J. & Flavian, S. (1964). *J. Chem. Soc.* pp. 2041–2051.

[bb5] Flack, H. D. (1983). *Acta Cryst.* A**39**, 876–881.

[bb6] Garnovskii, A. D., Nivorozhkin, A. L. & Minkin, V. I. (1993). *Coord. Chem. Rev.* **126**, 1–69.

[bb7] Henrici-Olive, G. & Olive, S. (1984). *The Chemistry of the Catalyzed Hydrogenation of Carbon Monoxide* Berlin: Springer.

[bb8] Ingold, C. K. (1969). *Structure and Mechanism in Organic Chemistry*, 2nd ed. Ithaca: Cornell University Press.

[bb9] Kılıç, I., Işık, Ş., Ağar, E. & Erşahin, F. (2009). *Acta Cryst.* E**65**, o1347. 10.1107/S1600536809018303 PMC2969673 21583199

[bb10] Köysal, Y., Işık, Ş. & Ağar, A. (2007). *Acta Cryst.* E**63**, o4916.

[bb11] Layer, R. W. (1963). *Chem. Rev.* **63**, 489–510.

[bb12] Şahin, Z. S., Işık, Ş., Erşahin, F. & Ağar, E.(2009). *Acta Cryst.* E**65**, o811. 10.1107/S1600536809009684 PMC2968928 21582533

[bb13] Sheldrick, G. M. (2003). *SADABS* University of Göttingen, Germany.

[bb14] Sheldrick, G. M. (2008). *Acta Cryst.* A**64**, 112–122. 10.1107/S0108767307043930 18156677

